# Performance of polychaete assisted sand filters under contrasting nutrient loads in an integrated multi-trophic aquaculture (IMTA) system

**DOI:** 10.1038/s41598-020-77764-x

**Published:** 2020-11-30

**Authors:** Daniel Jerónimo, Ana Isabel Lillebø, Andreia Santos, Javier Cremades, Ricardo Calado

**Affiliations:** 1 ECOMARE & CESAM, Departamento de Biologia, Universidade de Aveiro, Campus Universitário de Santiago, 3810-193 Aveiro, Portugal; 2 Coastal Biology Research Group (BioCost), Facultad de Ciencias & CICA, Universidade da Coruña, 15071 A Coruña, Spain

**Keywords:** Marine biology, Sustainability

## Abstract

Polychaete assisted sand filters (PASFs) allow to combine a highly efficient retention of particulate organic matter (POM) present in aquaculture effluent water and turn otherwise wasted nutrients into valuable worm biomass, following an integrated multi-trophic aquaculture (IMTA) approach. This study evaluated the bioremediation and biomass production performances of three sets of PASFs stocked with ragworms (*Hediste diversicolor*) placed in three different locations of an open marine land-based IMTA system. The higher organic matter (OM) recorded in the substrate of the systems which received higher POM content (Raw and Df PASFs – filtered raw and screened by drum filter effluent, respectively) likely prompted a superior reproductive success of stocked polychaetes (final densities 2–7 times higher than initial stock; ≈1000–3000 ind. m^−2^). Bioremediation efficiencies of ≈70% of supplied POM (≈1.5–1.8 mg L^−1^) were reported in these systems. The PASFs with lower content of OM in the substrate (Df + Alg PASFs – filtered effluent previously screened by drum filter and macroalgae biofilter) differed significantly from the other two, with stocked polychaetes displaying a poorer reproductive success. The PASFs were naturally colonized with marine invertebrates, with the polychaetes *Diopatra neapolitana*, *Terebella lapidaria* and *Sabella* cf. *pavonina* being some of the species identified with potential for IMTA.

## Introduction

Marine and brackish water aquaculture production contribute significantly for the world food security and in 2018 represented approximately 56% and 45% of the volume and value generated by this sector (values above 111 million tonnes and USD 250 billions)^1^. The production of fish contributed greatly for these values being reported productions of ≈12% and 31% of the volume and value of saltwater production in 2018 (diadromous species included)^1^. The intensive production of the majority of these organisms require well nutritionally balanced formulated feeds. The total use of aquafeeds estimated for 2016 alone was ≈49.6 million tonnes, being expected to rise to 76.2 million tonnes by 2025^2^. Not all these feeds are fully converted into biomass of cultured species and a non-negligible portion of these nutrients are often wasted in the form of uneaten feed, or due to the inability of farmed species to fully assimilate ingested nutrients (being commonly excreted through faeces)^3–6^. Some studies pointed that only 25–40% of the whole nitrogen and phosphorus (N and P, respectively) available in aquafeeds is truly assimilated in the form of biomass by fed species^6,7^. Carnivorous finfish excrete between 50 to 80% of feed N and 35 to 85% of feed P^8^. These nutrients are present in the water in the form of particulate organic matter (POM), dissolved organic matter (DOM) (include dissolved organic nitrogen [DON] and phosphorus [DOP]) and dissolved inorganic nutrients (include dissolved inorganic nitrogen [DIN] = NO_x_-N + NH_4_-N and dissolved inorganic phosphorus [DIP] = PO_4_–P)^9,10^. In this way, the investment made by producers in aquafeeds is not fully recovered in the form of biomass by the target species being farmed, and wasted nutrients commonly need to be eliminated from the productive process (including effluent water) by more or less complex processes that ultimately represent another financial burden. The recovery of these nutrients into valuable biomass and the consequent reduction of capital loss from uneaten aquafeeds (> 50% of operating costs^11^) are goals that can be achieved by adopting an integrated multi-trophic aquaculture (IMTA) approach. This concept considers the integrated production of commercially valuable species that rank in different levels of the trophic chain, in order to maximize the recovery of nutrients initially supplied through aquafeeds to the production system, but are not fully used by the target species being fed. This concept, and their main potentialities and limitations has been the topic of numerous reviews in recent years^12–19^. In marine land-based production, the recovery of valuable nutrients present in effluent waters can be pursued through the integration of extractive species capable of recovering available POM. Marine polychaetes have been often pinpointed as holding great potential to recover wasted nutrients from POM with species such as *Hediste diversicolor*^9,20–25^, *Perinereis aibuhitensis*^26^, *Alitta virens*^26,27^, *Perinereis nuntia*^28,29^, *Perinereis helleri*^29,30^*, Arenicola marina*^26^, *Abarenicola pusilla* ^31,32^*, Capitella* sp. and *Ophryotrocha craigsmithi*^33^ already being tested under IMTA designs. These marine worms have already been successfully combined with organisms that may retain dissolved inorganic nutrients efficiently, such as macro and microalgae^22,23^, as well as halophytes^9^.

The ragworm, *Hediste diversicolor* (O.F. Müller, 1776), has been a key species to include in IMTA models, as it allows the recovery of otherwise wasted nutrients in the form of a biomass rich in highly unsaturated fatty acids (HUFAs), namely eicosapentaenoic (EPA, 20:5 *n* − 3), docosahexaenoic (DHA, 22:6 *n* − 3) and arachidonic (ARA, 20:4 *n* − 6)^20–25^. The ability of this species to synthesise de novo polyunsaturated fatty acids (PUFAs) and HUFAs^34^ is an important feature when selecting the different trophic compartments that will be included in an IMTA design. The recovery of nutrients from aquafeeds into ragworms biomass is of great relevance if one considers the high demand for lipids and FAs (namely *n* − 3 HUFAs) for both human and animal nutrition^22^. Additionally, this species is also commonly used in marine finfish and crustaceans maturation diets^22,34^ and is one of the most prized baits for sports fishing^35^. The development of production models for these organisms allows to suppress their growing demand and avoid over-exploitation of natural stocks^36–38^. By reworking the substrate, ragworms can be termed as biodiffusors with important ecosystem engineering functions^39,40^. These organisms build extensive burrows and promote bioturbation (i.e. biogenic transport of sediment particles and pore water which destroys sediment stratigraphy^41^) and bioirrigation (i.e. ventilation of burrows and diffusion of oxidized solutes by infauna^41,42^). Microenvironments with steep gradients between reduced and oxidized compounds are created in polychaetes burrows, which act as transition zones that support enhanced microbial activities and are favour reoxidation processes^39,42,43^. Biogeochemical processes, such as carbon oxidation reactions (e.g. denitrification), manganese, iron and sulphate reduction are highly dependent on reoxidation and transport processes associated to bioturbation^39,42,44^.

Polychaete assisted sand filters (PASFs), are sand column filters stocked with marine worms that are highly efficient in the recovery of POM in the form of these species biomass^9,20,21,29,30^. By fostering the retention of POM and contributing to its mineralization, thus enhancing the availability of dissolved inorganic nutrients, PASFs can play a key role in IMTA designs including macro/micro algae and/or halophytes, if integrated as the first extractive unit^9,23^. In order to fine tune the use of PASFs in IMTA, the present work tested the performance of PASFs stocked with the ragworm *H. diversicolor* in different locations of an open marine land-based IMTA facility. These locations were selected to ensure that PASFs were supplied with effluent water with contrasting loads of nutrients, in order to better understand how these would limit or improve the successful production of ragworms. To achieve this goal a first set of PASFs was installed to filter the raw fish farm effluent originating from earthen ponds stocked with gilthead seabream (*Sparus aurata*); the second set to filter the same effluent but screened by a drum filter (45-µm mesh size) and finally the third set to filter the same effluent screened by a drum filter and subsequently by a macroalgae biofilter (stocked with sea lettuce, *Ulva rigida*).

## Results

### Characterization of inflowing water and POM bioremediation promoted by polychaete assisted sand filters (PASFs)

Table 1 summarizes the average values (± SD) of pH, concentration of dissolved oxygen (DO), temperature and salinity monitored in the inflowing water of each PASFs over 15 weeks at 10 AM, 2 PM and 6 PM (weekly characterization – Supplementary Fig. S1–S4). The PERMANOVA analysis revealed significant differences in the environmental conditions of inflowing water supplying each set of PASFs at 10 AM (*p* = 0.001), 2 PM (*p* = 0.001) and 6 PM (*p* = 0.001) (Supplementary Table S1). The SIMPER analysis (cut-off level 90%) revealed the existence of variable dissimilarities between, Raw – Df (4.2—7.9%), Raw – Df + Alg (10.2—12.8%) and Df – Df + Alg (6.3—7.3%), respectively. The following parameters that contributed the most for the dissimilarities recorded are summarized in Supplementary Table S2. The lowest average value of pH and oxygen were measured in Raw, while the lowest variance of both parameters between periods was recorded in Df system. The DO measured at 2 PM in Df + Alg PASFs was approximately twice that recorded at Raw PASFs.

**Table 1:** Average values (± SD) of pH, dissolved oxygen (DO), temperature and salinity measured weekly (n = 5) in the inflowing water of each set of polychaete assisted sand filters (PASFs) at 10 AM, 2 PM and 6 PM. Raw PASFs—received the raw effluent from the earthen pond stocked with *Sparus aurata*; Df PASFs—received the raw effluent after being screened by a drum filter; and Df + Alg PASFs—received effluent after being screened by a drum filter and a macroalgae biofilter stocked with *Ulva rigida*.

	10 AM				2 PM			6 PM		
PASFs	pH	O_2_ (mg L^−1^)	Temp. (C)	Salinity	pH	O_2_ (mg L^−1^)	Temp. (°C)	pH	O_2_ (mg L^−1^)	Temp. (°C)
Raw	7.66 ± 0.14	5.52 ± 0.94	19.28 ± 2.29	40.08 ± 1.01	7.64 ± 0.12	5.98 ± 0.66	20.96 ± 2.28	7.73 ± 0.17	6.47 ± 1.26	20.49 ± 4.00
Df	7.78 ± 0.15	7.95 ± 0.25	19.44 ± 2.28	40.07 ± 1.01	7.81 ± 0.16	8.01 ± 0.42	20.81 ± 2.44	7.83 ± 0.19	7.94 ± 0.77	20.31 ± 3.99
Df + Alg	8.20 ± 0.24	8.40 ± 0.80	18.87 ± 2.29	40.04 ± 1.11	8.61 ± 0.19	10.11 ± 0.94	21.27 ± 2.41	8.81 ± 0.14	8.68 ± 0.83	20.76 ± 2.97

**Figure 1:**
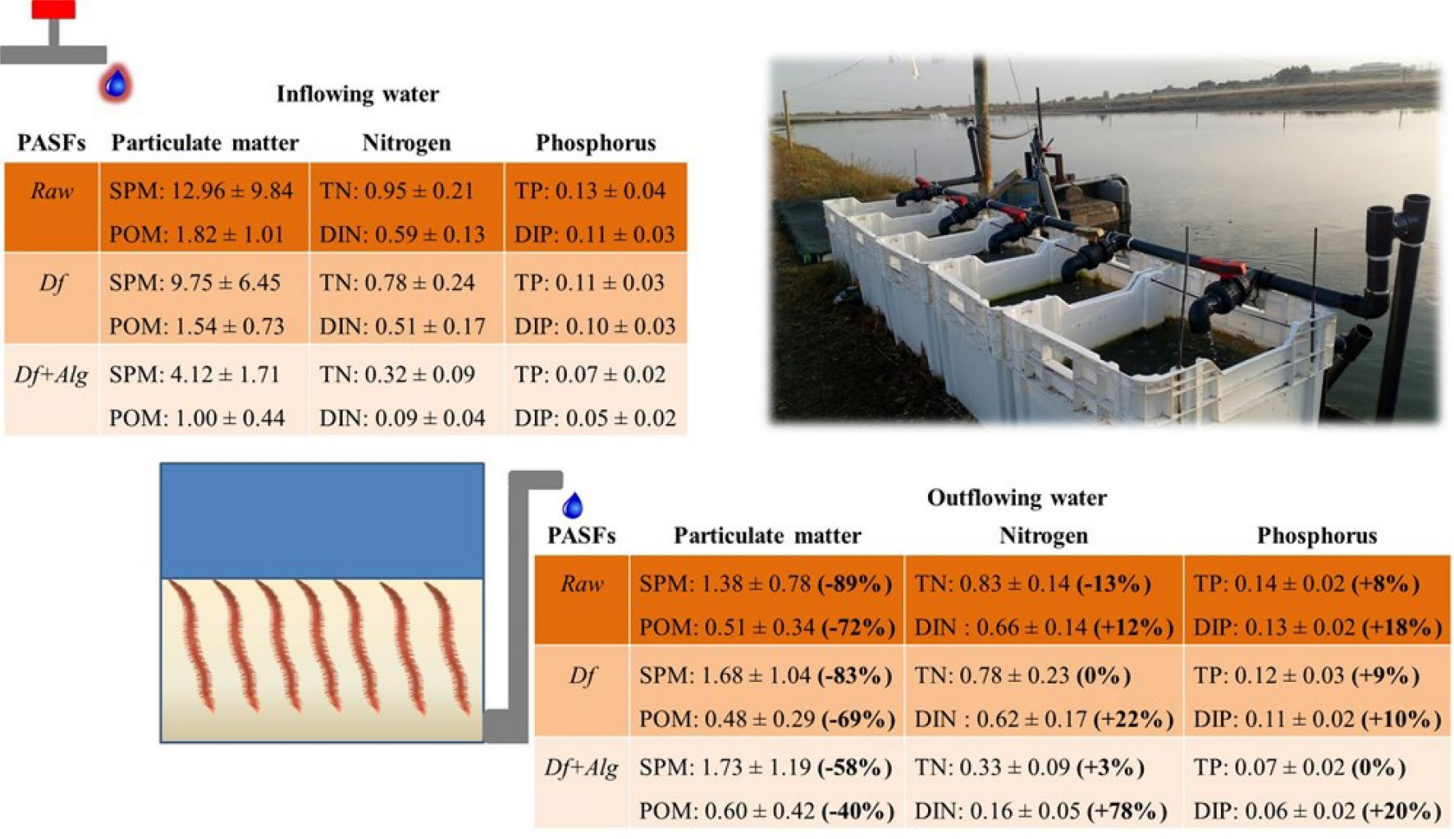
Average values (± SD) of suspended particulate matter (SPM), particulate organic matter (POM), total nitrogen (TN), dissolved inorganic nitrogen (DIN), total phosphorus (TP) and dissolved inorganic phosphorus (DIP) of the values determined over 15 consecutive weeks (n = 15) in each of the three sets of polychaete assisted sand filters (PASFs). Raw PASFs—received the raw effluent from the earthen pond stocked with *Sparus aurata*; Df PASFs—received the raw effluent after being screened by a drum filter; and Df + Alg PASFs—received effluent after being screened by a drum filter and a macroalgae biofilter stocked with *Ulva rigida*.

Figure 1 summarizes the average values (± SD) of suspended particulate matter and particulate organic matter (SPM and POM, respectively), total and dissolved inorganic nitrogen (TN and DIN, respectively) and total and dissolved inorganic phosphorus (TP and DIP, respectively) in inflowing and outflowing water of each PASFs. No significant differences were recorded in the composition of inflowing water supplying Raw and Df PASFs (PERMANOVA: *p* = 0.240) (Supplementary Table S3). SIMPER analysis (cut-off 90%) revealed dissimilarities of 8.35% (Supplementary Table S4). The composition of inflowing water supplying Df + Alg PASFs differed significantly from Raw PASFs (PERMANOVA: *p* = 0.001) and Df PASFs (PERMANOVA: *p* = 0.001). SIMPER analysis (cut-off 90%) revealed dissimilarities of 21% and 14.5%, respectively.

Raw, Df and Df + Alg PASFs promoted the retention of approximately 72%, 69% and 40% of supplied POM. These values represent removal efficiencies of 4.4, 3.5 and 1.3 mg L^−1^ m^−2^ (for Raw, Df and Df + Alg PASFs and refer to weekly characterizations—Supplementary Fig. S5). No significant differences were found for POM content present in the outflowing water of all PASFs (Kruskal–Wallis: *p* = 0.335) (Supplementary Table S5). During the study the concentration of OM (%LOI) at the sand bed of the PASFs was maintained between 0.42—0.69%, 0.63—0.75% and 0.36—0.58% (for Raw, Df and Df + Alg PASFs, respectively) and at the end of experiment no significant differences were recorded between the OM content of Raw and Df PASFs (Kruskal–Wallis: *p* = 0.917) (0.69 ± 0.03% and 0.75 ± 0.16%, respectively) (Supplementary Table S5). However, the content reported for Df + Alg (0.58 ± 0.05%) was significantly lower to that recorded in the other PASFs tested (Kruskal–Wallis: *p* = 0.009 and *p* = 0.028, respectively).

The outflowing water of each PASFs displayed higher levels of DIN than inflowing water (+ 12%, + 22% and + 78% for Raw, Df and Df + Alg PASFs, respectively). The predominant form of DIN in inflowing water was NH_4_-N, while in the outflowing water of PASFs the most abundant form was NO_x_-N (sum of NO_2_-N and NO_3_-N) (Supplementary Fig. S6 and S7, respectively). Concerning phosphorus, the outflowing water presented higher concentrations of TP (+ 8%, + 9%, + 0%, for Raw, Df and Df + Alg PASFs, respectively) and DIP (+ 18%, + 10% and + 20%, for Raw, Df and Df + Alg PASFs, respectively) compared to the concentrations in the inflowing water.

### Biomass generation

**Figure 2:**
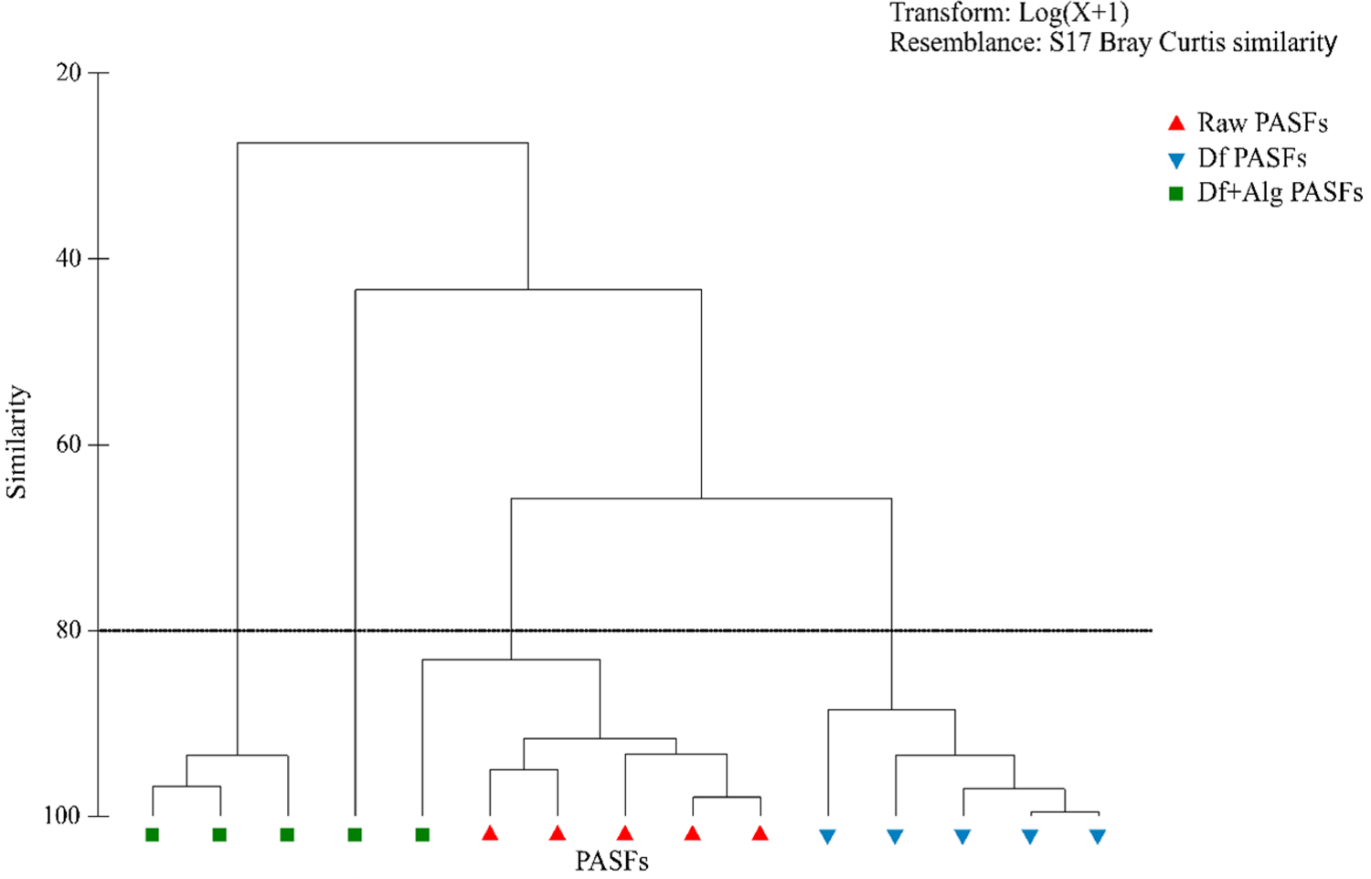
CLUSTER analysis of *H. diversicolor* groups composition (initially stocked and newly generated specimens) recorded in each polychaete assisted sand filter (PASFs). Raw PASFs—received the raw effluent from the earthen pond stocked with *Sparus aurata*; Df PASFs—received the raw effluent after being screened by a drum filter; and Df + Alg PASFs—received effluent after being screened by a drum filter and a macroalgae biofilter stocked with *Ulva rigida*.

Table 2 summarizes the average density (± SD) of *H. diversicolor* determined in each PASFs at the end of experimental period (15 weeks). No significant differences were found between final densities of Raw and Df PASFs (Kruskal–Wallis: *p* = 0.117) (Supplementary Table S6). These PASFs presented average values of 996 ± 627 and 3015 ± 2485 ind. m^−2^ (respectively), which corresponded to ≈ 2—sevenfold increases of the initial stocking density. The final density reported for Df + Alg PASFs was significantly lower (79 ± 64 ind. m^−2^) from that recorded for the other two sets of PASFs (Kruskal–Wallis: *p* = 0.009 and *p* = 0.009, respectively), with ≈ sixfold decrease of initial stocking density. In respect to the proportion between initially stocked polychaetes and newly generated ones, 90% and 100% of the specimens identified in the Raw and Df PASFs (respectively) were classed as newly generated biomass (< 5 mm). Most specimens identified in Df + Alg PASFs corresponded to adult polychaetes (≈ 86%) belonging to the initial stock. Figure 2 displays the cluster analysis of *H. diversicolor* group composition (initially stocked and newly generated specimens) which allow to verify that all the replicates of Raw and Df PASFs were represented in separate and well-defined groups, with a similarity between them higher than 88%. In Df + Alg PASFs, three of the replicates do not present any signs of reproduction (Supplementary Table S7) and therefore exhibited less than 30% similarity with other PASFs. The biomass of *H. diversicolor* recorded at the end of the experiment was 1.2 ± 0.8, 0.3 ± 0.2 and 0.6 ± 0.8 g Ash-Free Dry Weight (AFDW) m^−2^ for Raw, Df and Df + Alg PASFs, respectively.

**Table 2:** Average values (± SD) of density (ind. m^−2^) and biomass (g. AFDW m^−2^) of *H. diversicolor* determined at each polychaete assisted sand filters (PASFs) at the end of experimental period. Raw PASFs—received the raw effluent from the earthen pond stocked with *Sparus aurata*; Df PASFs—received the raw effluent after being screened by a drum filter; and Df + Alg PASFs—received effluent after being screened by a drum filter and a macroalgae biofilter stocked with *Ulva rigida*.

PASFs	Original stock	New generation	Total	
	Density (ind. m^−2^)	Density (ind. m^−2^)	Density (ind. m^−2^)	Biomass (g. AFDW m^−2^)
Raw	100 ± 59	896 ± 642	996 ± 626 ^a^	1.16 ± 0.80
Df	ND	3015 ± 2485	3015 ± 2485 ^a^	0.28 ± 0.22
Df + Algae	73 ± 51	18 ± 25	91 ± 55 ^b^	0.63 ± 0.82

**Figure 3:**
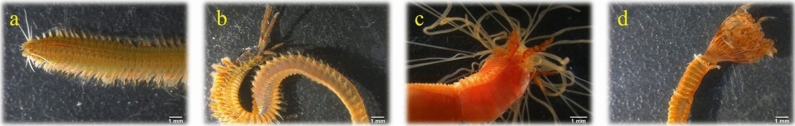
Polychaete species presented in polychaete assisted sand filters (PASFs): (**A**) *Hediste diversicolor*; (**B**) *Diopatra neapolitana*; (**C**) *Terebella lapidaria* and (**D**) *Sabella* cf. *pavonina*.

The three sets of PASFs were exposed to a potential colonization of other species occurring in the coastal lagoon supplying the earthen pond stocked with seabream. Table 3 summarizes the densities and biomass of the most well-represented species in each PASFs, with emphasis to polychaetes *Diopatra neapolitana* (Onuphidae), *Terebella lapidaria* (Terebellidae) and *Sabella* cf. *pavonina* (Sabellidae) (represented in Fig. 3). The total biomass (all species included) accounted for approximately 8.4, 6.1 and 5.2 g AFDW m^−2^ at the Raw, Df and Df + Alg, respectively.

**Table 3:** Biomass (g. AFDW m^−2^) and density (ind. m^−2^) of the most represented species (excluding *Hediste diversicolor*) present in different polychaete assisted sand filters (PASFs). Raw PASFs—received the raw effluent from the earthen pond stocked with *Sparus aurata*; Df PASFs—received the raw effluent after being screened by a drum filter; and Df + Alg PASFs—received effluent after being screened by a drum filter and a macroalgae biofilter stocked with *Ulva rigida*.

PASFs	Density (ind. m^−2^)	Biomass (g AFDW m^−2^)
**Raw**		
*Capitella capitata*	7415 ± 2017	1.63 ± 0.73
*Terebella lapidaria*	4237 ± 941	2.80 ± 0.28
*Chorophium* sp.	2906 ± 1513	0.16 ± 0.11
*Melita palmata*	1476 ± 861	0.48 ± 0.30
*Hydrobia acuta*	1177 ± 326	0.42 ± 0.12
*Sabella* cf. *pavonina*	444 ± 117	1.20 ± 0.52
*Phallusia mammillata*	308 ± 137	0.56 ± 0.30
*Actiniaria* sp.	163 ± 109	0.40 ± 0.41
*Diopatra neapolitana*	82 ± 74	0.73 ± 0.97
**Df**		
*Capitella capitata*	3468 ± 1083	0.61 ± 0.27
*Terebella lapidaria*	2327 ± 556	2.85 ± 0.98
*Hydrobia acuta*	1286 ± 640	0.95 ± 0.97
*Actiniaria* sp.	353 ± 152	0.17 ± 0.06
*Phallusia mammillata*	199 ± 76	1.16 ± 0.91
*Sabella* cf. *pavonina*	81 ± 74	0.36 ± 0.41
**Df + Alg**		
*Hydrobia acuta*	5586 ± 2056	1.63 ± 0.40
*Boccardia polybranchia*	2363 ± 796	0.25 ± 0.12
*Haminoea* sp.	1087 ± 1245	1.06 ± 0.67
*Malacoceros* sp.	1014 ± 840	0.16 ± 0.12
*Terebella lapidaria*	290 ± 214	1.95 ± 1.19
*Phallusia mammillata*	45 ± 45	0.18 ± 0.24

## Discussion

In the present work, the three sets of PASFs successfully recovered POM present in effluent waters in the form of valuable worm biomass. The Raw and Df PASFs, which filtered the raw effluent water from the production ponds of gilthead seabream and the same effluent but previously screened by a drum filter, respectively, retained approximately 70% of POM (1.8 and 1.5 mg L^−1^, respectively). The lowest efficiency in POM retention (≈ 40% of 1.0 mg L^−1^) was displayed by Df + Alg PASFs, most likely due to these tanks receiving smaller-sized particulate matter. This prevalence of smaller-sized particles resulted from the joint action of mechanical filtration (which fragmented larger-sized particles) and the deposition of larger particles in the macroalgae biofilter. It is also important to highlight that the nature of POM present in Df + Alg PASFs was certainly different from that in other PASFs, mostly resulting from the macroalgae biofilter (essentially macroalgae biomass) instead of fish feed/faeces. The use of the different systems tested will filter approximately 2000 L m^−2^ day^−1^. Based on filtering efficiencies recorded, POM retention will vary between 2.1—2.6 g m^−2^ day^−1^ using Raw and Df PASFs, as long as the composition of the inflowing water is maintained throughout the day. A lower efficiency is expected to occur for Df + Alg PASFs (0.8 g m^−2^ day^−1^).

To prevent the build-up of OM, and consequently preserve the filter function of sand bed, it is paramount that polychaetes successfully secure bioturbation and bioirrigation processes^9,45^. In a previous study also employing *H. diversicolor* in sand beds to filter the effluent derived from super-intensive production of Senegalese sole (*Solea senegalensis*), up to 70% of OM was removed after a 24-weeks trial^9^. In this previous study, PASFs secured a higher filtering rate (4320 L m^−2^ day^−1^) than that reported in the present work (2000 L m^−2^ day^−1^). PASFs stocked with polychaetes *Perinereis nuntia* and *P. helleri* to filter a shrimp farm effluent (culturing *Penaeus monodon*) were able to reduce SPM by 50% at a flow rate similar to that used in the present study^29^.

Regarding the effect of PASFs in the dynamics of dissolved inorganic nutrients, it was recorded that these promoted the mineralization of OM, thus increasing the concentrations of DIN and DIP, a process that had already been reported in previous studies^9,29^. By employing these sand filters stocked with polychaetes the same level of nitrogenous and organic compounds degradation can be obtained as when employing other filtration systems more commonly used in aquaculture (e.g. plastic biological ball filters)^46^. The high efficiency in POM retention and the contribution to enhance the bioavailability of dissolved inorganic nutrients (DIN and DIP) makes PASFs an appealing option for IMTA designs. Indeed, this approach allows to consider the integration of a second extractive unit receiving the outflowing water and targeting the uptake of dissolved inorganic nutrients (e.g. by using marine macro/micro algae or halophytes). A complete design integrating PASFs (*H. diversicolor*) and halophytes in aquaponics (*Halimione portulacoides*) was already successfully tested^9^, with the second extractive unit being able to recover 67% of the DIN present in the outflowing water of PASFs. The polychaete *H. diversicolor* had already been tested to filter the water of a RAS under a complete IMTA design that also included macroalgae biofilters (*Ulva lactuta* or *Solieria chordalis*)^23^. The results obtained in the present study reinforce the biomitigation potential of *H. diversicolor* when aiming to impair the loss of nutrients available in the effluents of fish farms. Indeed, at the end of the experimental period (15 weeks) only the Raw and Df PASFs that presented the highest OM content in their sand beds also displayed a high reproductive success of stocked polychaetes (90–100% of specimens with a size < 5 mm). The lower reproductive success recorded for polychaetes stocked in Df + Alg PASFs may partly be explained by the higher fluctuations in the pH and oxygen recorded in inflowing water, as well as a much lower input of POM. The final densities of polychaetes recorded for Raw and Df PASFs were lower than the ones reported in previous studies (e.g. 7000 ind. m^−2^ using and initial stocking density of ≈ 400 ind. m^−2^ after 24 weeks)^9^. This difference may be explained by the shorter duration of the present study, as well as by the lower nutrient loads present in the effluent water being supplied to the PASFs. The full harvesting of the fish being farmed, and the consequent loss of IMTA conditions, made it impossible to extend the study period beyond 15 weeks. As such the evaluation of generated biomass was mostly performed based in small sized polychaetes larvae (< 5 mm), as the reproductive behaviour of *H. diversicolor* results in the death of mature worms (monotelic species)^47^. It is therefore recommended that the evaluation of growth and biomass generation of this polychaete should be performed over a longer period after the initial stocking with adult biomass (> 5 months). This procedure will guarantee a correct stabulation, reproduction and growth of new cohorts of polychaetes. Another approach is to use nectochaetes to initially stock PASFS, as this would likely allow a faster evaluation of growth performances and biomass generation. This strategy has already been successfully used in a previous study, where PASFs were stocked with juvenile forms of *Perinereis helleri* and *P. nuntia* and productivities of approximately 300—400 g FW m^−2^ were reported after ≈ 5 months^29^.

Other polychaete species naturally colonizing the PASFs, such as *Diopatra neapolitana*, *Terebella lapidaria*, and *Sabella* cf. *pavonina*, adapted very well to the culture conditions. These polychaetes may eventually be tested in future trials featuring them as extractive species in IMTA. While *D. neapolitana* already presents a well-defined market potential, as it is commonly collected to be used as bait in sports fishing^48^, the potential use of *Sabella* cf. *pavonina* and *T. lapidaria* is yet to be evaluated. It is worth highlighting that *T. lapidaria*, was able to successfully adapt to each of the three PASFs tested, being the average dry weight (AFDW) recorded for this species 10 times higher in the Df + Alg PASFs. This result was likely due to the lower specific richness recorded and subsequent lower trophic competition. Overall, the present findings allow us to conclude that the best locations to position PASFs stocked with *H. diversicolor* were Raw and Df PASFs positions, systems which showed the best efficiency in retaining POM into valuable polychaete biomass. The PASFs also favoured biogeochemical processes to increase the concentration of DIN and DIP thus revealing a potential to enhance the growth of micro/macroalgae and halophyte plants positioned in subsequent extractive units.

## Material and methods

### Selected extractive species

The polychaete *Hediste diversicolor*, popularly known as ragworm, was selected for the present study due to its wide distribution along the shallow marine and brackish waters of European and North American estuaries and by being an infaunal species that produce three-dimensional burrow network in sandy-mud bottoms^49^. This species is classified as presenting free movement via the burrow system and as a biodiffusor in the sediment reworking, thus presenting an important action in bioturbation and bioirrigation—the biogenic modification of sediments through particle reworking and burrow ventilation, a key mediating process of many important geochemical processes in marine systems^40^. This polychaete species is omnivorous, being classified as an active predator^50^. However, it also exhibits a deposit-feeding behaviour that allows it to mainly consume organic matter present in the substrate^47,51^. The two main feeding strategies it displays are crawling on the sediment surface prospecting for food, catching it with its jaws and ingesting it immediately, as well as capturing food with mucous secretions that are deposited at the entrance of its burrow^47^. Bacteriolytic activity in their digestive tract demonstrates that this species is a significant bacteriovore as well^52^. Juveniles can accumulate plant detritus in their burrow where constant irrigation holds aerobic conditions that favour the decay process of plant debris by stimulating bacterial growth^53^. Ragworms can also be facultative filter-feeders, which meet metabolic requirements on a pure diet of phytoplankton, much like a typical obligate filter-feeder species^54,55^. Its life cycle is characterized by females brooding their embryos in the maternal burrow, where its short pelagic larval life takes place^47^. The environmental engineering behaviour, along with the fact of exhibiting a biomass rich in essential fatty acids (EFA) makes them an appealing extractive species for IMTA systems.

### IMTA experimental design

**Figure 4:**
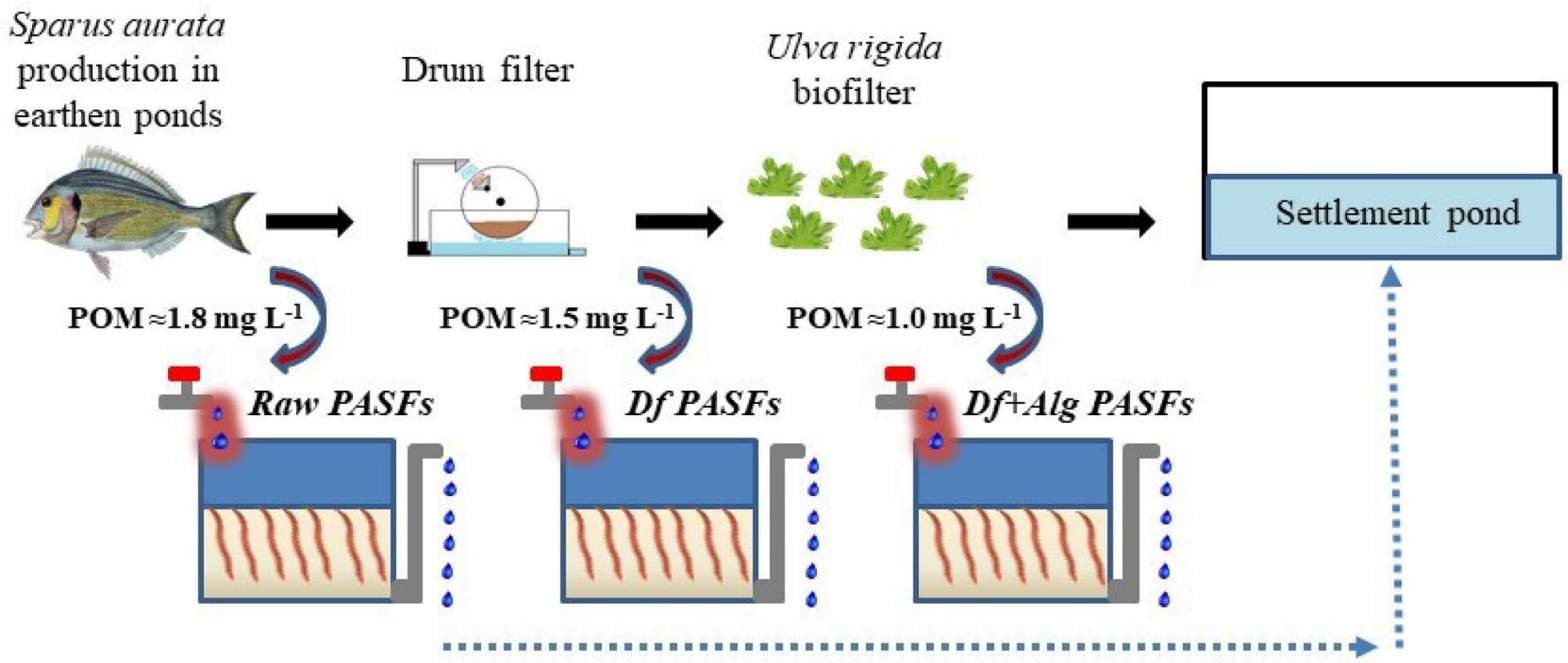
Schematic representation of the experimental set-up adopted with polychaete assisted sand filters (PASFs) placed in different locations of an open marine land-based IMTA facility: Raw PASFs—received the raw effluent from the earthen pond stocked with *Sparus aurata*; Df PASFs—received the raw effluent after being screened by a drum filter; and Df + Alg PASFs—received effluent after being screened by a drum filter and a macroalgae biofilter stocked with *Ulva rigida*.

The organic rich effluent used in the present study resulted from the semi-intensive production of gilthead seabream (*Sparus aurata*) (≈ 12.000 specimens with average weight ≈ 400 g) stocked in an earthen pond and fed twice a day (SFR ≈ 1.5% day^−1^) with a commercial diet that present 43% of crude protein, 17% of crude fat and 10% of crude fiber (Standard Orange 4; AQUASOJA). The effluent water used was collected at the end of this earthen pond. The first set of PASFs was supplied with the raw effluent water without any type of filtration (Raw PASFs), while the second set received the raw effluent but mechanically filtered by a drum filter (45 µm mesh size) (Df PASFs). The third set received the raw effluent filtered by the drum filter and after passing through a macroalgae biofilter stocked with *Ulva rigida* (Df + Alg PASFs). The algae biofilter presented a volume of 36 m^−3^ (surface area of 24 m^2^), with a flow rate varying between 50 and 100 L h^−1^ (3.3–7% renewal day^−1^) and *U. rigida* being cultured at a density between 2.5 – 5 kg FW m^−2^. Each of the above-mentioned sets of PASFs consisted of 5 tanks each arranged in a parallel set-up. Each replicate tank from each of the three sets of PASFs presented a volume of 0.1 m^3^ and a surface area of 0.3 m^2^ and featured a 200 mm bottom sand bed (0.7—1 mm grain size) and a superficial 100 mm water column. To allow a complete percolation of the effluent water being supplied through the sand bed, each tank was equipped with a bottom draining pipe bellow the sand bed. Each tank received an effluent flow of 25 L h^−1^ (0.5 renewal each hour) and the treated water was not re-used, thus being the system employed an open-IMTA. The schematic representation of the experimental set-up adopted is presented in Fig. 4. The present study was run for a total of 15 weeks (from July 2017 to November 2017), during which no additional feed was provided to any of the three sets of PASFs.

### IMTA extractive species cultivation

Wild specimens of *H. diversicolor* were collected in Aveiro coastal lagoon by local fisherman and each sand bed was inoculated with an initial density of 440 ind. m^−2^ (167 g m^−2^) of polychaetes with a length superior to 40 mm. Fifteen weeks post-stocking, the polychaetes biomass was evaluated by performing five hand core samples (Ø 75 mm, 150 mm depth) from each replicate tank on each of the three sets of PASFs, with their content being preserved in buffered 4% formaldehyde for latter analysis. In the laboratory, specimens of *H. diversicolor* were sorted in two distinct groups (new generation biomass, displaying a length < 5 mm and original stocking biomass, with a length > 40 mm). Other polychaete species that naturally colonised the PASFs were also sorted and identified to species level according to Fauvel (1923)^56,57^. Ash free dry weight (AFDW) of *H. diversicolor* and other polychaete species that naturally colonized the PASFs were determined by loss of ignition method (LOI%; 5 h combustion at 450 ºC of samples previously dried at 90 ºC, until constant weight was recorded). Sediment samples from each replicate tank of each of the three sets of PASFs were collected in triplicate at the beginning and at the end of experiment to determine organic matter (OM) content. This content was determined by the difference between dry weight and ash free dry weight, using the LOI% determination described above.

### IMTA monitoring

Temperature, pH, dissolved oxygen (DO) and salinity determined in the inflowing water of each of the three sets of PASFs was monitored weekly. Due to differences promoted by seaweed biofilter during daytime, each parameter was monitored at three distinct periods: 10 AM, 2 PM and 6 PM. The monitoring was performed using a multi-parameter probe Lovibond SensoDirect 150. Samples from the inflowing water of each of the five tanks of the three sets of PASFs, as well as the outflowing water of each PASFs tank after having percolated through the sand bed, were collected every week, in order to determine suspended particulate matter (SPM), particulate organic matter (POM), total nitrogen (TN), total phosphorus (TP), dissolved inorganic nitrogen (DIN = NOx-N + NH_4_-N) and dissolved inorganic phosphorus (DIP = PO_4_-P). Water samples were transported to the laboratory under dark and refrigerated conditions and immediately filtered (Whatman GF/C, Ø47mm dehydrated (105 °C) and pre-weighed filters) and subsequently frozen (− 20 °C) until further analysis. Filters containing SPM were processed following the EPA method 160.2 (USEPA) and POM was determined by loss of ignition method (LOI%; 5 h combustion at 450 ºC of samples previously dried at 90 ºC, until constant weight was recorded), resulting from the difference between dry weight and ash free dry weight (AFDW). Water samples were analysed using an automated continuous flow analyser (Skalar San^++^) to determine the content of TN, TP, NH_4_-N and PO_4_–P. The oxidized forms of NOx-N were determined using a flow injection system (FIAstar 5000 Analyser). The analytical quality control was ensured by using calibration curves that result from running standard solutions at the beginning and in parallel with blanks and samples. All analyses were performed according to the protocols made available to each parameter by the equipment´s manufacturer.

### Statistical analysis

Data retrieved over 15 consecutive weeks on the environmental parameters (Temp., oxygen, pH, salinity) of the inflowing water supplying each of the three sets of PASFs being compared (Raw, Df and Df + Alg PASFs) were used to prepare three independent resemblance matrixes. A first resemblance matrix was prepared for environmental parameters being monitored in triplicate at 10 AM (n = 3), a second one for parameters monitored at 2 PM (n = 3) and a third matrix for parameters monitored at 6 PM (n = 3). The rationale for assembling these three independent resemblance matrixes was the shifts known to occur a priori on the environmental parameters of the inflowing water caused by time of day. Each of these three resemblance matrixes was compared separately using permutational multivariate analysis of variance (PERMANOVA), with PASFs being used on each of them as a fixed predictive factor (with three levels: Raw, Df and Df + Alg). A fourth resemblance matrix was also prepared using data retrieved over 15 consecutive weeks on the following parameters of the inflowing water being supplied to each of the three sets of PASFs: SPM, POM, TN, DIN, TP and DIP. Samples were always collected in triplicate (n = 3) at 10 AM. This resemblance matrix was also compared using PERMANOVA, with PASFs also being used as a fixed predictive factor (with three levels: Raw, Df and Df + Alg). All resemblance matrixes were prepared using Euclidean distances of data previously normalized. Whenever significant differences (*p* < 0.05) were detected, these were further examined using a posteriori pair-wise comparison. Similarity percentage (SIMPER) analysis (cut-off 90%) were also performed to evaluate the percentage that each environmental parameter (Temp., oxygen, pH, salinity) or water composition parameters (SPM, POM, TN, DIN, TP and DIP) contributed to the dissimilarity recorded between PASFs. PERMANOVA and SIMPER analysis were performed using PRIMER v6 with the PERMANOVA + add-on (PRIMER-E, UK), according to the procedures described by Anderson, Gorley & Clarke (2006)^58^.

To compare POM retention efficiency in each of PASFs tested, the level of POM present in the outflowing water of each of the five tanks (n = 5) from the three sets of PASFs being compared (Raw, Df and Df + Alg PASFs) were determined over 15 consecutive weeks at 10 AM. The existence of significant differences was tested using the non-parametric test of Kruskal–Wallis (*p* < 0.05) with PASFs being used as fixed predictive factor (with three levels). The organic matter recorded in the sand beds of each of the five tanks (n = 5) from the three sets of PASFs being compared at the end of experiment, as well as differences in the final abundance (ind. m^−2^) of *H. diversicolor*, were compared between each pair of PASFs using the non-parametric test of Kruskal–Wallis (*p* < 0.05). Data were previously checked for normality (Anderson–Darling test) and homogeneity of variances (Bartlett´s and Levene´s tests). These statistical analyses were performed using Minitab 18 Statistical Software (State College, PA).

A cluster analysis of the ratio between initial stocking and abundance of newly generated *H. diversicolor* recorded in each tank was also performed using PRIMER v6 with the PERMANOVA + add-on (PRIMER-E, UK).

The statistical results of the tests mentioned above are summarized in supplementary Tables S1–S6.

## Supplementary information

Supplementary Information.

## Data Availability

All data generated or analysed during this study are included in this published article and its Supplementary Material files.
