# Supplementary material for: Performance of polychaete assisted sand filters under contrasting nutrient loads in an integrated multi-trophic aquaculture (IMTA) system
Source: Sci Rep. 2020 Nov 30;10:20871. doi: 10.1038/s41598-020-77764-x (PMC7705650; doi:10.1038/s41598-020-77764-x)
Supplement: Supplementary file 1 — Supplementary Information. [file 41598_2020_77764_MOESM1_ESM.pdf]

Supplementary Information for

Performance of polychaete assisted sand filters under contrasting nutrient loads in an integrated multi-trophic aquaculture (IMTA) system

Daniel Jerónimo*, Ana Isabel Lillebø, Andreia Santos, Javier Cremades, Ricardo Calado*

*Corresponding authors:

E-mail: danieljeronimo@ua.pt; Tel.: +351938547866 (D Jerónimo)

E-mail: rjcalado@ua.pt; Tel.: +351234370779 (R Calado)

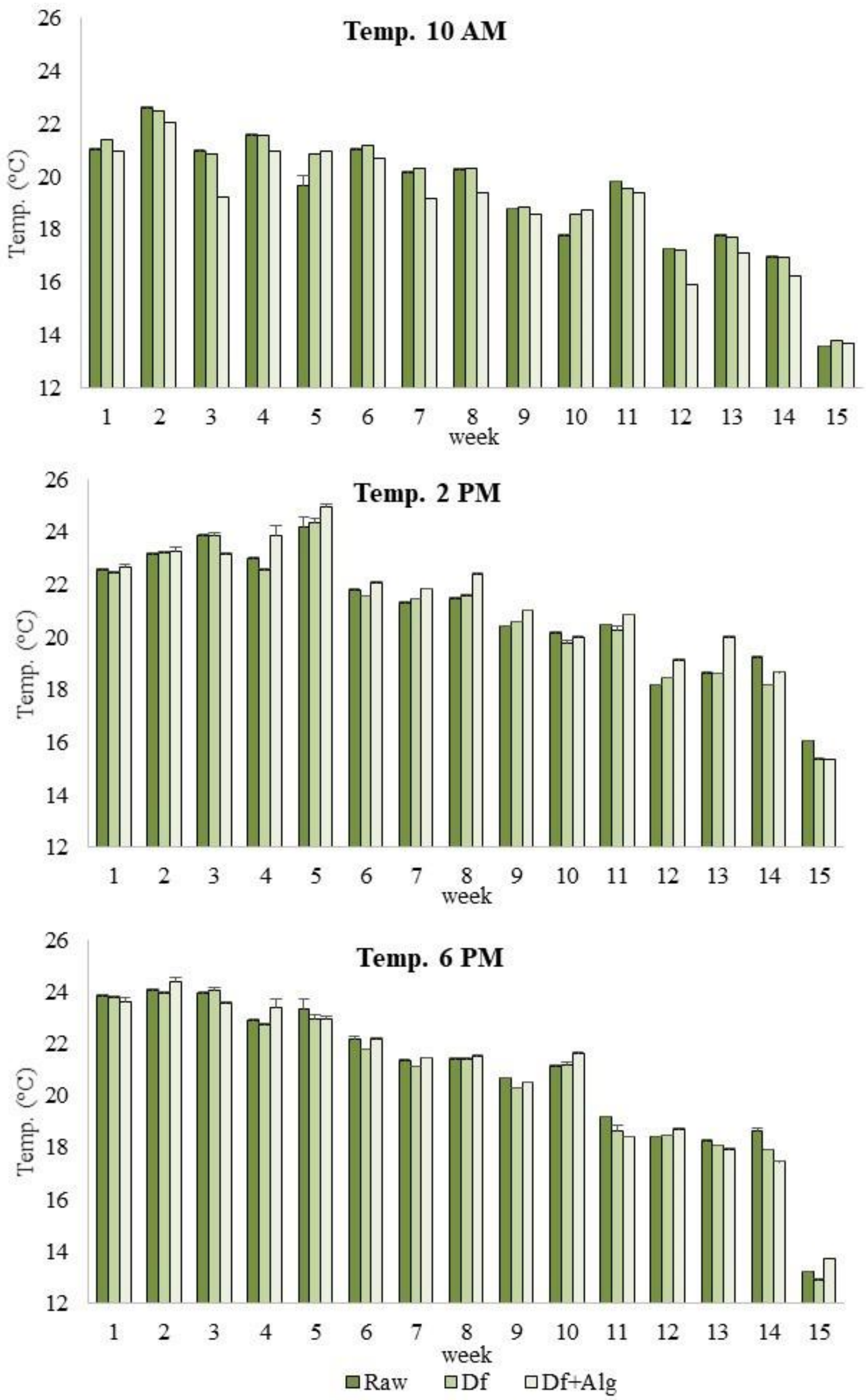

Figure S1. Weekly characterisation of temperature monitored in the inflowing water of each polychaete assisted sand filter (PASFs) at 10 AM, 2 PM and 6 PM. Average values (±SD) (n=3).

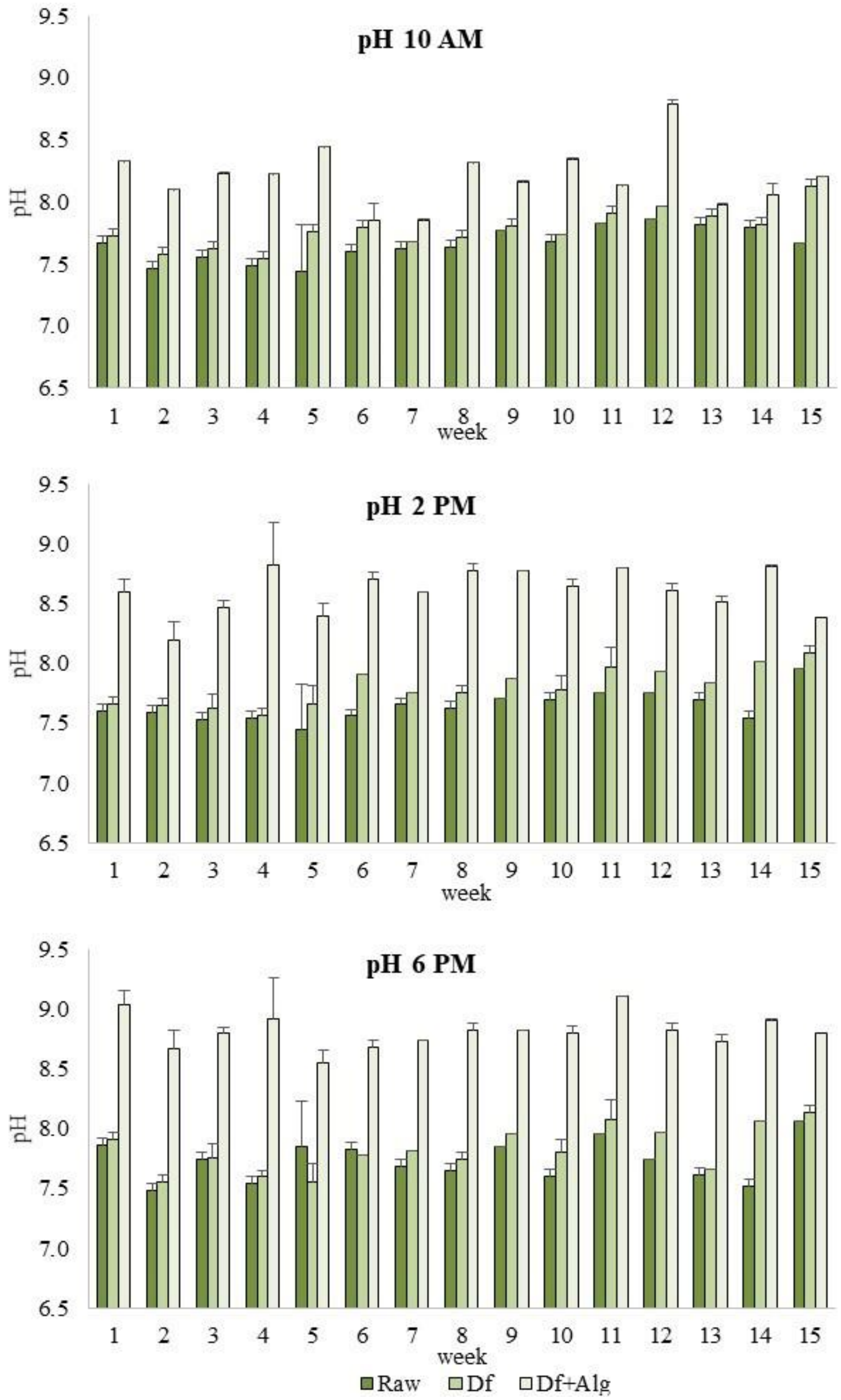

Figure S2. Weekly characterisation of pH monitored in the inflowing water of each polychaete assisted sand filter (PASFs) at 10 AM, 2 PM and 6 PM. Average values (±SD) (n=3).

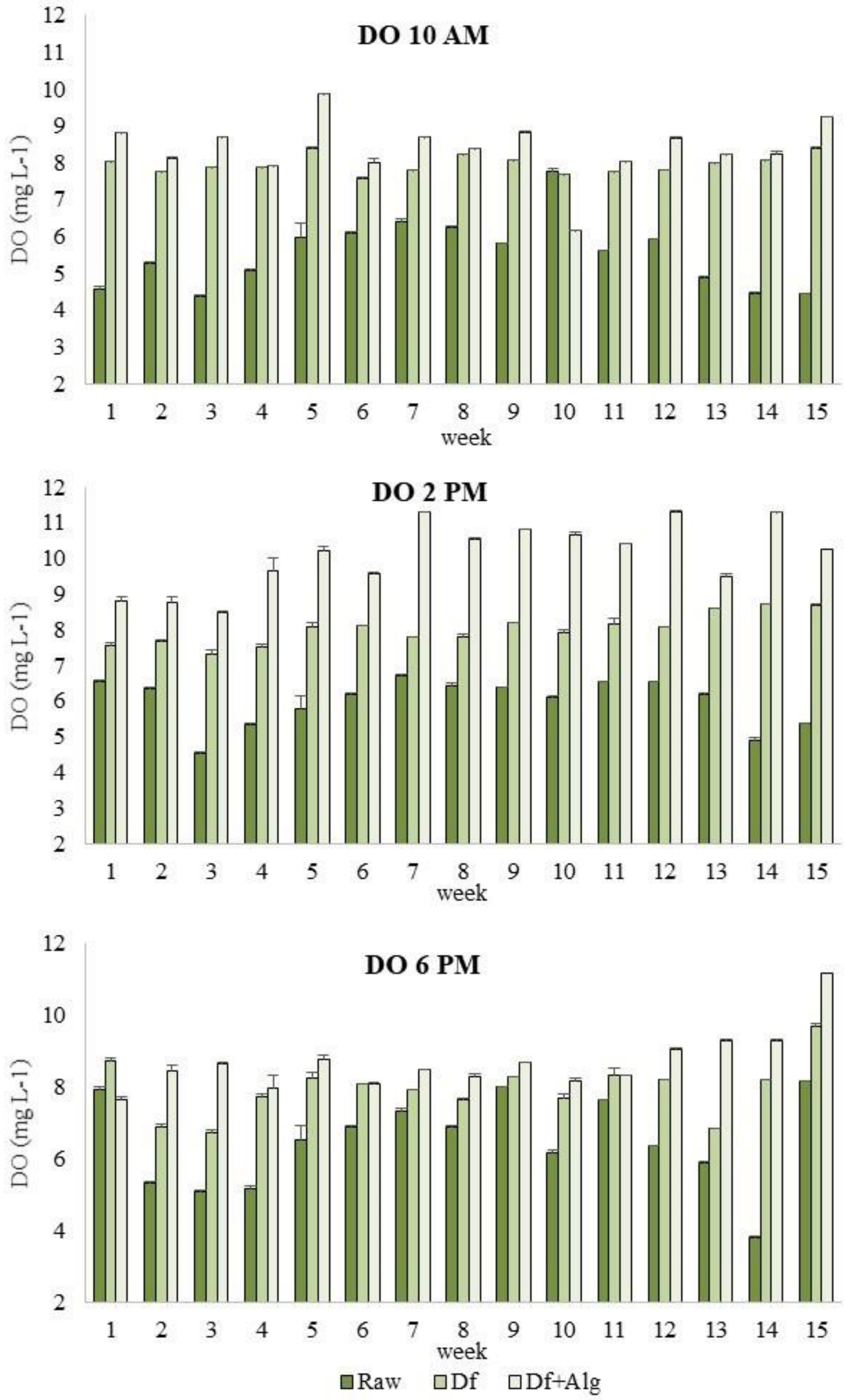

Figure S3. Weekly characterisation of dissolved oxygen (DO) concentration monitored in the inflowing water of each polychaete assisted sand filter (PASFs) at 10 AM, 2 PM and 6 PM. Average values (±SD) (n=3).

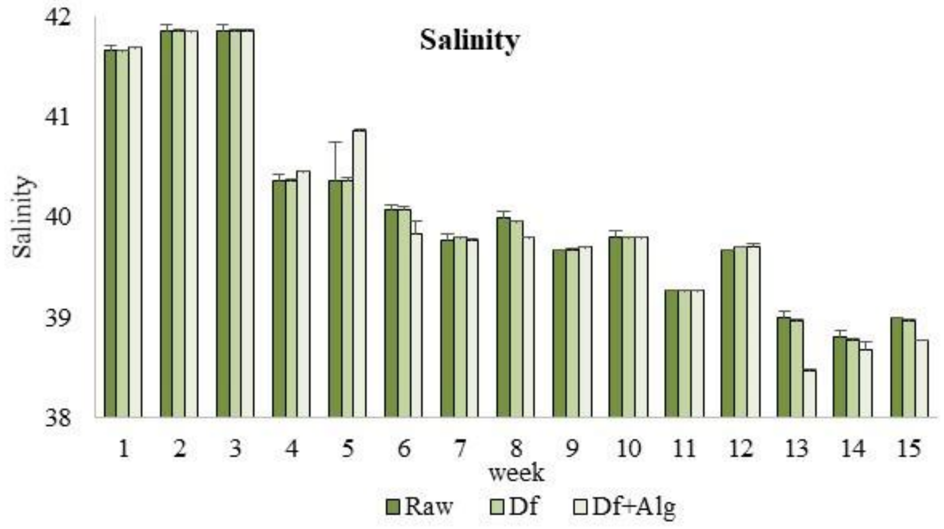

Figure S4. Weekly characterisation of salinity measured in the inflowing water of each polychaete assisted sand filter (PASFs). Average values (±SD) (n=3).

Table S1. Permutational multivariate analysis of variance (PERMANOVA) to evaluate variations in the environmental parameters (Temp., oxygen, pH, salinity) monitored in inflowing water between different polychaetes assisted sand filters (PASFs) in each of the monitored periods: 10 AM, 2 PM and 6 PM. Significant differences when $p<0.05$

	*Pseudo-F and t-value**	*P(perm)*
**10AM**		
Global Test		
PASFs	35.670	0.001
Pairwise Tests		
Raw - Df	5.240*	0.001
Raw - Df+Alg	7.632*	0.001
Df - Df+Alg	4.365*	0.001
**2PM**		
Global Test		
PASFs	90.291	0.001
Pairwise Tests		
Raw - Df	5.028*	0.001
Raw - Df+Alg	12.844*	0.001
Df - Df+Alg	8.677*	0.001
**6PM**		
Global Test		
PASFs	56.330	0.001
Pairwise Tests		
Raw - Df	3.837*	0.001
Raw - Df+Alg	9.892*	0.001
Df - Df+Alg	8.044*	0.001

Table S2. Similarity percentage analysis (SIMPER) (cut-off 90%) to evaluate contributions of each parameters to dissimilarities between polychaetes assisted sand filters (PASFs) in each of the monitored periods: 10 AM, 2 PM and 6 PM.

**10 AM**			**2 PM**			**6 PM**		
Raw & Df			Raw & Df			Raw & Df		
Avg. Dissimilarity = 7.9			*Avg. Dissimilarity = 4.2*			*Avg. Dissimilarity = 5.5*		
Parameter	Contrib. (%)	Cum. (%)	Parameter	Contrib. (%)	Cum. (%)	Parameter	Contrib. (%)	Cum. (%)
O_2	43.4	43.4	*T*	47.0	47.0	O_2	48.8	48.8
T	24.7	68.1	O_2	44.8	91.8	*T*	46.0	94.8
Sal	23.4	91.5						
Raw & Df+Alg			Raw & Df+Alg			Raw & Df+Alg		
Avg. Dissimilarity = 12.8			*Avg. Dissimilarity = 12.1*			*Avg. Dissimilarity = 10.2*		
Parameter	Contrib. (%)	Cum. (%)	Parameter	Contrib. (%)	Cum. (%)	Parameter	Contrib. (%)	Cum. (%)
O_2	34.8	34.8	O_2	44.3	44.3	*pH*	44.4	44.4
pH	33.3	68.2	*pH*	39.8	84.2	O_2	38.7	83.1
Sal	16.0	84.2	*T*	15.8	100	*T*	16.9	100
T	15.8	100						
Df & Df+Alg			Df & Df+Alg			Df & Df+Alg		
Avg. Dissimilarity = 7.3			*Avg. Dissimilarity = 6.7*			*Avg. Dissimilarity = 6.3*		
Parameter	Contrib. (%)	Cum. (%)	Parameter	Contrib. (%)	Cum. (%)	Parameter	Contrib. (%)	Cum. (%)
pH	40.0	40.0	*pH*	50.8	50.8	*pH*	60.9	60.9
Sal	28.3	68.3	*T*	31.4	82.1	*T*	27.5	88.4
T	27.7	96.0	O_2	17.9	100	O_2	11.6	100

Table S3. Permutational multivariate analysis of variance (PERMANOVA) to evaluate variations in the inflowing water composition (SPM, POM, TN, DIN, TP and DIP) between different polychaetes assisted sand filters (PASFs). Significant differences when $p<0.05$

	*Pseudo-F and t-value**	*P(perm)*
Global Test		
PASFs	17.984	0.001
Pairwise Tests		
Raw - Df	1.201*	0.240
Raw - Df+Alg	5.817*	0.001
Df - Df+Alg	5.363*	0.001

Table S4. Similarity percentage analysis (SIMPER) (cut-off 90%) to evaluate contributions of each parameters to dissimilarities verified in composition of water supplied to different polychaetes assisted sand filters (PASFs).

Raw & Df			Raw & Df+Alg			Df & Df+Alg		
Avg. Dissimilarity = 8.4			*Avg. Dissimilarity = 21*			*Avg. Dissimilarity = 14.5*		
Parameter	Contrib. (%)	Cum. (%)	Parameter	Contrib. (%)	Cum. (%)	Parameter	Contrib. (%)	Cum. (%)
POM	24.6	24.6	*DIN*	20.7	20.7	*DIN*	23.2	23.2
SPM	23.4	47.9	*TN*	20.4	41.1	*TN*	19.2	42.4
TP	17.2	65.2	*TP*	16.9	58.0	*DIP*	16.6	58.9
DIP	16.9	82.1	*DIP*	16.3	74.3	*TP*	15.5	74.5
TN	11.2	93.2	*SPM*	13.5	87.7	*SPM*	13.3	87.8
			POM	12.3	100	*POM*	12.2	100

Table S5. Kruskal-Wallis test to evaluate variations in particulate organic matter (POM) monitored in outflowing water and to evaluate variations in organic matter (OM) content monitored in sand beds between different polychaetes assisted sand filters (PASFs). Significant differences when *p*<0.05

	p-value
**POM monitored in outflowing water between PASFs**	
Global Test	
PASFs	0.335
**OM recorded in sand bed between PASFs**	
Global Test	
PASFs	0.018
Pairwise Tests	
Raw - Df	0.917
Raw - Df+Alg	0.009
Df - Df+Alg	0.028

Table S6. Kruskal-Wallis test to evaluate variations in density (ind. m^{-2}) of *Hediste diversicolor* determined in each polychaete assisted sand filters (PASFs) at the end of experimental period (15 weeks). Significant differences when *p*<0.05

	p-value
**Density of *H. diversicolor***	
Global Test	
PASFs	0.005
Pairwise Tests	
Raw - Df	0.117
Raw - Df+Alg	0.009
Df - Df+Alg	0.009

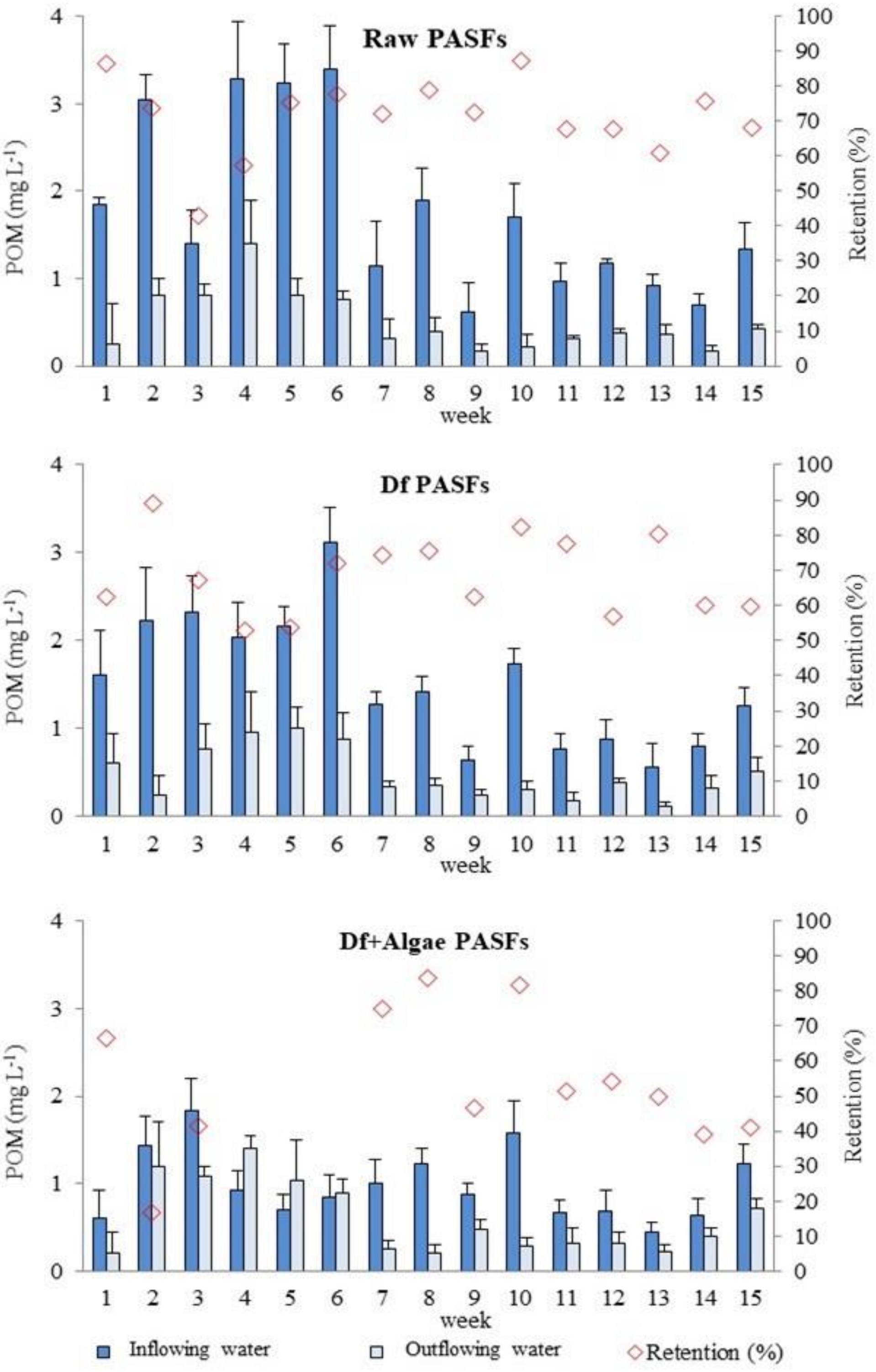

Figure S5. Weekly characterisation of particulate organic matter (POM) measured in the inflowing and outflowing water of Raw, Df and Df+Alg polychaete assisted sand filters (PASFs). The red diamond represent the percentage of retention (left axis; difference between inflowing and outflowing) in PASFs. Average values (±SD) (n=5).

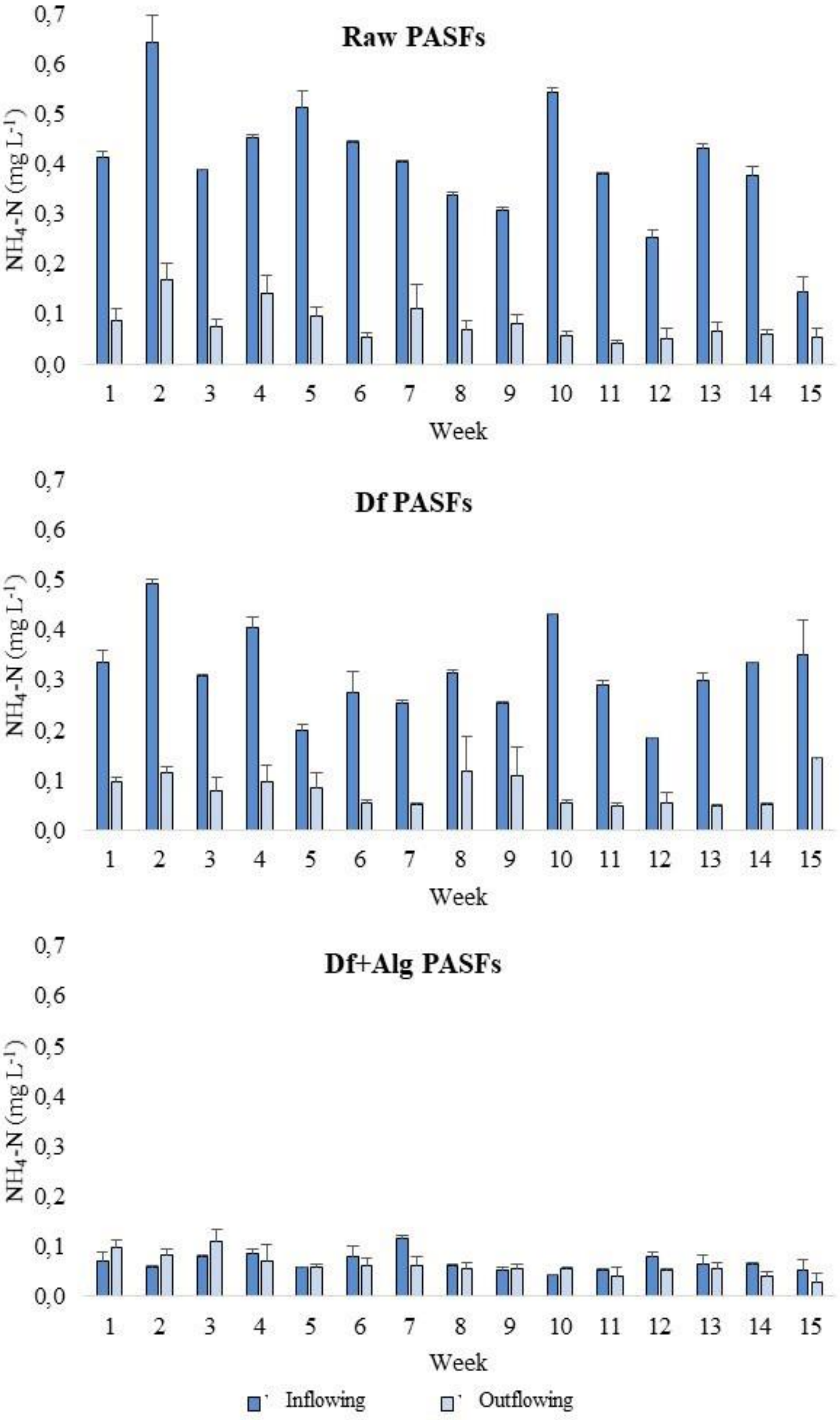

Figure S6. Weekly characterisation of ammonium-nitrogen (NH_4-N) monitored in the inflowing and outflowing water of Raw, Df and Df+Alg polychaete assisted sand filters (PASFs). Average values (±SD) (n=5).

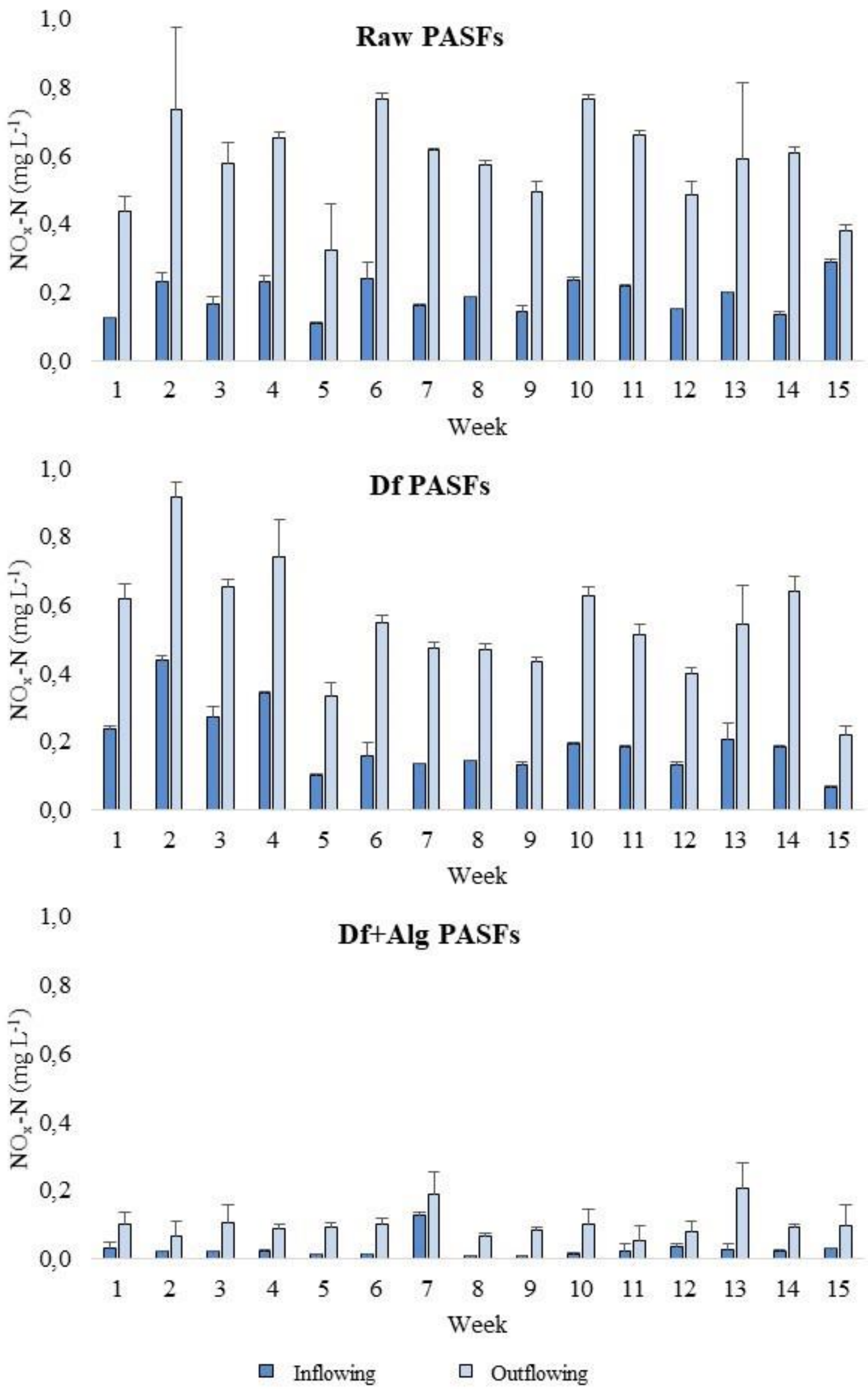

Figure S7. Weekly characterisation of oxidized forms of dissolved inorganic nitrogen (NOx-N) monitored in the inflowing and outflowing water of Raw, Df and Df+Alg polychaete assisted sand filters (PASFs). Average values (±SD) (n=5).

Table S7. Density of *H. diversicolor* (ind. m^{-2}) determined at each replicate (Tk) of polychaete assisted sand filters (PASFs) at the end of experimental period.

	Raw PASFs					Df PASFs					Df+Alg PASFs				
Group	Tk 1	Tk 2	Tk 3	Tk 4	Tk 5	Tk 1	Tk 2	Tk 3	Tk4	Tk 5	Tk 1	Tk 2	Tk 3	Tk 4	Tk 5
Initial stock (ind. m^{-2})	181	45	91	45	136	ND	ND	ND	ND	ND	91	ND	91	45	136
New generation (ind. m^{-2})	452	407	1901	1177	543	1765	2897	2671	589	7153	ND	45	ND	ND	45
Total (ind. m^{-2})	633	453	1992	1222	679	1765	2897	2671	589	7153	91	45	91	45	181
